# Effectiveness of an integrative programme in reducing hypertension incidence among the population at risk for hypertension: A community-based randomized intervention study in Shanghai, China

**DOI:** 10.7189/jogh.12.11013

**Published:** 2022-12-17

**Authors:** Jiayun Wang, Qiyun Jiang, Dan Gong, Honglian Liu, Peng Zhou, Donglan Zhang, Xing Liu, Jun Lv, Chengyue Li, Huiqi Li

**Affiliations:** 1 Department of Health Policy and Management, School of Public Health, Fudan University, China; 2 Research Institute of Health Development Strategies, Fudan University, China; 3 Changning District Xinhua Street Community Health Service Center, China; 4 Changning District Center for Disease Control and Prevention, China; 5 Division of Health Services Research, Department of Foundations of Medicine, New York University Long Island School of Medicine, USA; 6 Department of Epidemiology, School of Public Health, Fudan University, China; 7 School of Public Health and Community Medicine, Institute of Medicine, University of Gothenburg, Sweden

## Abstract

**Background:**

We aimed to evaluate the effectiveness of a community-based integrative programme in reducing hypertension incidence among populations at high risk for hypertension in Shanghai, Eastern China.

**Methods:**

We conducted a cluster-randomized intervention trial with a total of 607 participants (intervention, n = 303; control, n = 304) between October 2019 and October 2020. A total of 605 participants (intervention, n = 302; control, n = 303) completed the follow-up survey. The intervention group received an integrative programme that included health education, physician follow-up, and self-management, while the control group received usual care only. We used questionnaires to investigate risk factors, knowledge, attitudes, and behaviours regarding hypertension prevention for all participants at baseline and follow-up. We measured the incidence of hypertension according to the predefined protocol based on the national definition during the four follow-ups (only applicable to the intervention group) and the physical examination at the end of the intervention/programme/study. The difference-in-difference (DID) effects of the intervention were estimated using Generalized Estimating Equations.

**Results:**

There were no significant differences in age group, gender, and educational level between intervention and control groups at baseline. The integrative programme reduced the incidence of hypertension in the intervention group compared to the control group (odds ratio (OR) = 0.27, 95% confidence interval (CI) = 0.12-0.61). The DID analysis found that the one-year intervention has improved the level of hypertension-related knowledge and attitudes regarding diagnostic criteria, complications of hypertension, and lifestyle modification (*P* < 0.05). The intervention was also associated with a 3.7% increase in the behaviour change rate of “not smoking” (OR = 2.50, 95% CI = 1.45-4.30) and a 34.8% increase in the rate of “monitoring blood pressure regularly” (OR = 29.61, 95% CI = 13.02-67.35).

**Conclusions:**

The integrative programme could reduce the risk for hypertension and improve the level of hypertension-related knowledge and attitudes, affecting the formation of healthy behaviours in high-risk populations. The community-based management for high-risk groups should be scaled up and incorporated into national hypertension control programmes, which may potentially reduce the substantial burden of hypertension and cardiovascular disease in China.

**Registration:**

ISRCTN registration number: ISRCTN74154693.

The population at risk for hypertension is a group with prevalent risk factors or characteristics and a higher risk of hypertension than normotensive people [1]. The global prevalence of individuals with high risk for hypertension in people aged 15 years and above was 38% in 2012 [2], while a 2013 meta-analysis found the prevalence in mainland China to be 37% in the last decade [3]. This population has a 2-fold risk of developing hypertension, and higher morbidity and mortality attributable to cardiovascular and cerebrovascular diseases compared to individuals who are not at risk [4,5]. The lack of health literacy and the prevalent unhealthy lifestyles have largely contributed to the growth of the population at risk for hypertension [6].

Health management of the at-risk population could be effective in delaying or preventing disease progression to hypertension [7,8]. Integrated interventions, including physical activity [9], dietary interventions [7], and health education [10], are evidence-based approaches to managing these high-risk individuals [4]. In China, communities play an important role in the management of the population at risk for hypertension, as primary care physicians establish a good relationship with community dwellers and carry out health education and health promotion activities [11], and are proven to be the most cost-efficient care delivery model [12]. For those efficacious integrative interventions to have a public health impact, they need to be implemented in community settings [13,14].

Several studies have evaluated the effects of community-based integrative interventions for managing the population at risk for hypertension, including self-monitoring of blood pressure (BP), dietary adjustments, and regular exercise. Some studies compared the changes in the indicators before and after the intervention, and others used quasi-experimental designs to evaluate the effects by comparing the intervention and control groups. For example, studies in the United States [15] and Thailand [16] have found promising results of such interventions in reducing BP levels. In China, high-quality evidence is lacking regarding the effects of community-based integrative interventions on the population at risk for hypertension. A few previous studies conducted in the Northern [17], Southern [18], and Eastern [19,20] regions of China suffered from small sample sizes or did not have a rigorous intervention trial design. There is a need to fill in this knowledge gap.

We aimed to analyse the effects of a community-based integrative programme on the incidence of hypertension and hypertension-related knowledge, attitudes, and behaviours (KAB) among the population at risk for hypertension using a cluster randomized controlled trial in a district of Shanghai in China. The results could provide stronger evidence for implementing a comprehensive intervention for managing the high-risk population to reduce the burden of hypertension and the rising incidence of cardiovascular disease in China.

## METHODS

### Study design

We conducted a community-based randomized intervention trial among the population at risk for hypertension in 2019, with measurements obtained at baseline and after a one-year follow-up to evaluate the effect of the integrative intervention. We selected a community in Changning District of Shanghai, located in the Eastern Region of China, as the study setting. In 2019, its gross domestic product per capita was 237 800 RMB (US$34 481), with 17 neighbourhood blocks and more than 80 000 residents. Six neighbourhood blocks were randomly selected for inclusion in the study, in which three blocks were randomized as the intervention group and the other three were assigned to the control group.

### Study participants

A multi-stage, cluster sampling was conducted to select the participants at risk for hypertension (the detailed inclusion/exclusion criteria are described in the next paragraph), based on the screening tool (concerning age, family history of hypertension, having diabetes, having dyslipidaemia, body mass index, and having abdominal obesity, with the area under the receiver operating characteristic curve being 0.817) [21] designed by our research team. We considered the awareness rate of hypertension-related knowledge as the key indicator for determining the sample size, assuming that the average awareness rate of the intervention group and the control group were 70% and 50%, respectively. A sample size of 280 for both the intervention and control groups was determined based on the confidence level (1 − α = 0.95), power (1 − β = 0.9), design effect (Deff = 2), and response rate of the survey (90%). Thus, around 1000 residents were needed to screen the required high-risk individuals, assuming that the prevalence of the population at risk for hypertension was about 30% [22]. We performed the sampling in the intervention group as follows: we randomly selected one lane from each sampled neighbourhood block by using a computer-generated random number, with approximately 300 to 400 residents in each lane. We invited all adult residents in the three sampled lanes to participate in our study. Excluding those who were absent during screening or not willing to participate in the screening, we screened 1089 residents using our screening tool to reach the required sample size. Similarly, we recruited the participants in the control group after screening 1109 residents.

**Figure 1:**
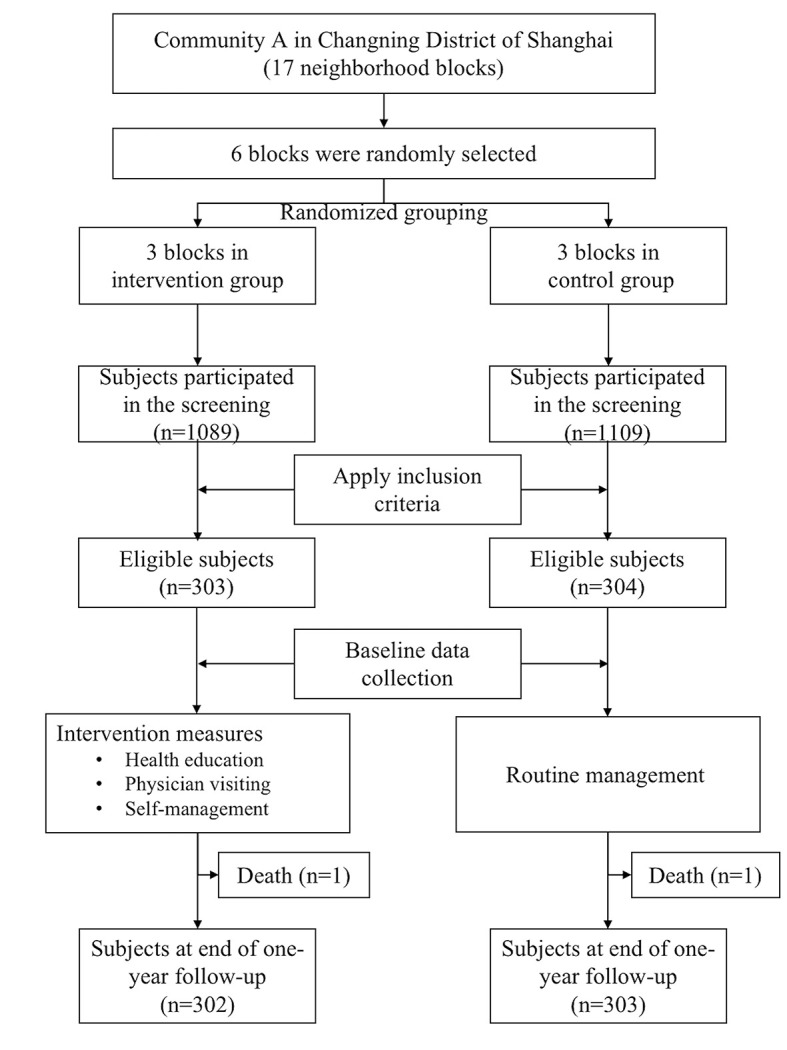
Study design and flow of participants inclusion in the cluster randomized controlled trial.

The inclusion criteria were: 1) residents aged 18-80 years of the sampled lane who have resided in the study area for more than half a year; 2) having been identified as individuals at risk for hypertension, with the total risk score more than 20 based on our screening tool [21]; 3) informed consent and willingness to participate in this study; 4) no previous history of hypertension diagnosis or use of anti-hypertensive medication; 5) with good cognitive function and physical condition. A total of 303 and 304 individuals at risk for hypertension were included in the intervention and control group at baseline, respectively. The total number of subjects at the end of the one-year follow-up for the intervention and control group were 302 and 303 individuals, as one participant in each group dropped out of the study due to death (**Figure 1**).

This study was approved by the Medical Research Ethics Committee at the School of Public Health of Fudan University, Shanghai, China (approval number: IRB#2017-TYSQ-03-10). All respondents gave informed consent.

### Intervention methods

We implemented the study from October 2019 to October 2020. All participants were enrolled in this study simultaneously in October 2019. The intervention group was managed through the integrative intervention and the control group received the usual care (ie, the general practitioners followed up with patients and queried their BP levels and lifestyles via phone every six months). A single-blind design was used in the intervention, with the participants blinded to the intervention status. The integrative programme consisted of three sections: health education, physician follow-ups, and self-management **(****Table 1****)**.

**Table 1:** Intervention measures and frequency in the intervention group

Session	Interventions	Major intervention content	Method	Frequency
1	Health education	Diagnostic criteria, causes, and characteristics of hypertension in adults; hazards and complications of hypertension; measurement method of BP; monitoring for the risk factors of hypertension and lifestyle intervention methods	Holding health education lectures	Once per quarter (ie, at the end of 3, 6, 9, and 12 mo after enrolment)
			Distributing health education leaflets	Once per month
			Releasing health-related knowledge using social media	Once per month
2	Physician follow-ups	Basic information about the participant (height, weight, systolic BP, diastolic BP, etc.); progress of risk factors of hypertension: changes in healthy behaviours	Group visits, online visits, telephone visits, and home visits	Once per quarter
3	Self-management	Changes in physical activity, diets, smoking, alcohol consumption, weight control, etc	Filling out self-management cards by the participants and getting feedback from the GPs	Once per month

BP – blood pressure, GPs – general practitioners, mo – months

Before the intervention, training was provided to all general practitioners (GPs) and staff involved in the intervention group regarding the intervention measures for high-risk individuals, the intervention contents, the implementation procedures, and rules for filling out the tables on the intervention process. The participants in the intervention and the control groups received the questionnaire surveys and physical examinations at the baseline, including blood pressure and hypertension-related KABs (see “Data collection” section for details). We allocated health education lecture schedules, self-management cards, and health education leaflets to individuals in the intervention group and created an online group chat using social media (WeChat, Tencent Co. Ltd, China). During the follow-up periods, GPs provided intervention services to the intervention groups per the integrative programme. At the end of the one-year follow-up, the intervention and the control groups received the questionnaire surveys and physical examinations again.

All recordings and documents of the management process were employed to ensure that assessment and intervention procedures were standardized across the study sites and all participants. Senior researchers routinely monitored the interviews, physical assessment procedures, and programme implementation activities.

### Data collection

Questionnaire surveys and physical examinations were conducted by trained GPs for participants in the two groups at baseline and one-year follow-up. All investigators were trained based on the contents of the questionnaire and interview techniques before the survey. The structured questionnaire consisted of four sections. Section 1 included sociodemographic characteristics, such as gender, age, educational level, and annual income per capita. Sections 2 to 4 included knowledge about hypertension prevention (9 items), the attitude toward hypertension prevention (6 items), and behavior and lifestyle change (6 items). Answering options in the latter three sections were dichotomous, ie, “Know/Don't Know” or “Yes/No”. The questionnaire was tested to have high internal consistency, with a Cronbach’s alpha coefficient reaching 0.838, 0.883, and 0.621 for the knowledge, attitudes, and behaviors sections, respectively [1].

Physiological data were measured following the standard procedure, including BP, height, weight, and waist circumference. BP was measured following the standard procedure recommended by the Chinese Guidelines for the Prevention and Treatment of Hypertension. After resting quietly in a seated position for 5 minutes, 3 consecutive BP readings were obtained by the automated validated Omron electronic sphygmomanometer (OMRON Corporation, Kyoto, Japan) using appropriate cuff size determined from an upper arm circumference measurement.

The completed questionnaire and records were checked by the research team. In case of any error or missing, the investigators would contact the subject by telephone to correct or add the information.

### Variable definitions

The primary outcome was the incidence of hypertension. During the four physician follow-ups (for the intervention group) and the end of the whole intervention (for both the intervention and control group), participants’ B*P* values were measured in physical examinations. According to the Chinese Guidelines for the Prevention and Treatment of Hypertension, if someone’s BP value was higher than normal (ie, they had a systolic BP of 140 mm Hg or higher or a diastolic BP of 90 mm Hg or higher), the GPs would measure the BP once a day for the next two days. If all three B*P* values exceeded the normal, an incident case of hypertension was ascertained. The secondary outcomes included changes in hypertension-related KAB, calculated by the difference in the formation rate of KAB between the baseline and the end of the intervention.

The recommended daily intake (RDI) of salt for adults was based on the Chinese Dietary Guidelines (no more than six grams per day). Current smokers were defined as those who were smoking and consuming at least one cigarette a day during the last six months. Current drinkers were defined as those who drank alcohol daily or occasionally. Control of salt intake was defined as intaking salt less than six grams daily. Controlling body weight was defined as measuring body weight and calculating body mass index (BMI) at least once per three months [23]. Frequent physical activity was defined as exercising more than four times per week and at least 30 minutes each time [24]. Regular BP monitoring was defined as measuring BP at least once per three months.

### Statistical analysis

We performed descriptive analyses to show the counts and percentages of categories of the participant characteristics. We conducted χ^2^ tests to compare the baseline demographic characteristics between the intervention and control groups and changes in the outcomes before and after the intervention within the intervention and control group. Given that the study was cluster randomized and each participant answered the questionnaire twice, the difference-in-difference (DID) effects of the intervention were estimated using generalized estimating equations, with consideration of the clustering effect of the neighbouring block. All models were adjusted for age, gender, educational level (high school or below/vocational college or above), and income groups (annual income per capita <40 000 RMB/40 000 ~ 60 000 RMB /60 000 ~ 80 000 RMB/≥80 000 RMB), and KAB at baseline were further included in the adjustment when analysing the effect on the incidence of hypertension. Odds ratios (ORs) and 95% confidence intervals (95% CIs) were obtained from the models. All tests were two-sided and *P* < 0.05 was considered statistically significant. All analyses were performed using SPSS 23.0 (SPSS Inc., Chicago, IL, USA) and Stata 16.0 (StataCorp LLC, Texas, USA).

## RESULTS

### Baseline characteristics

The enrolled participants’ general characteristics (303 individuals in the intervention group and 304 individuals in the control group) at baseline are presented in **Table 2**. There were no significant differences in age group, gender, and educational level between these two groups (*P* > 0.05), while significant differences in income groups between these two groups (*P* < 0.001) were observed.

**Table 2:** Socio-demographic characteristics between the intervention and control groups at baseline

Variables	Intervention group (n = 303)	Control group (n = 304)	*P*-value*
**Age (n (%))**			
<50 y	46 (15.2)	51 (16.8)	0.844
50-64 y	97 (32.0)	93 (30.6)	
≥65 y	160 (52.8)	160 (52.6)	
**Gender (n (%))**			
Male	151 (49.8)	149 (49.0)	0.840
Female	152 (50.2)	155 (51.0)	
**Educational level (n (%))**			
High school or below	218 (71.9)	227 (74.7)	0.448
Vocational college or above	85 (28.1)	77 (25.3)	
**Annual income per capita (n (%))**			
<40 000 RMB	15 (5.0)	64 (21.0)	<0.001
40 000-60 000 RMB	80 (26.4)	95 (31.3)	
60 000-80 000 RMB	108 (35.6)	86 (28.3)	
≥80 000 RMB	100 (33.0)	59 (19.4)	

*P-values were calculated by the χ^2^ tests.

### Intervention effects on the incidence of hypertension

The incidence of hypertension in the intervention and control group at one-year follow-up was 2.3% and 10.6%, respectively (**Table 3**). The DID analysis showed that the odds of developing hypertension in the intervention group were 75% lower than that in the control group (OR = 0.25, 95% CI = 0.10-0.60). Further adjustments for KAB at baseline did not substantially change the effect estimates (OR = 0.27, 95% CI = 0.12-0.61).

**Table 3:** Intervention effects on the incidence of hypertension for the intervention and control group at one-year follow-up analysed as difference-in-difference (DID) effects

				Model 1		Model 2	
**Variable**	**Intervention group (n, %)**	**Control group (n, %)**	**DID (%)**	**OR**	**95% CI**	**OR**	**95%CI**
Baseline	0 (0)	0 (0)	-8.3	0.25	0.10-0.60	0.27	0.12-0.61
One-year follow-up	7 (2.3)	32 (10.6)					
*P*-value	0.008*	<0.001*		0.002†		0.002‡	

DID – difference-in-difference, CI – confidence interval, OR – odds ratio

*P-values were calculated by χ^2^ tests.

†Model 1 was adjusted for age, gender, educational level, and income.

‡Model 2 was adjusted additionally for KAB at baseline.

### Intervention effects on the levels of hypertension-related knowledge, attitudes, and behaviours

The intervention effects on hypertension-related KAB for the intervention and control group at one-year follow-up are presented in **Table 4**. Regarding hypertension-related knowledge, the rates of correct answers on “hypertension is a lifelong disease” (75.2% vs 93.0%), “diagnostic criteria of hypertension” (51.5% vs 79.8%), “hypertension is related to high salt intake” (80.9% vs 90.7%), “coronary heart disease is a complication of hypertension” (57.8% vs 88.7%), “stroke is a complication of hypertension” (75.9% vs 86.1%), and “RDI of salt for adults” (64.0% vs 94.7%) significantly improved at the one-year follow-up in the intervention group. However, in the control group, only knowledge of “RDI of salt for adults” changed significantly, with rates at baseline and one-year follow-up being 57.6% and 69.3%, respectively.

**Table 4:** Intervention effects on hypertension-related knowledge, attitudes, and behaviours for the intervention and control group at one-year follow-up analysed as difference-in-difference (DID) effects

Items	Intervention group			Control group			DID (%)	OR (95% CI)†	*P*-value
	**Baseline (n (%))**	**Follow-up (n, %)**	*P*-value *	**Baseline (n (%))**	**Follow-up (n, %)**	*P*-value *			
**Knowledge of hypertension prevention**									
Whether hypertension is a life-long disease	228 (75.2)	281 (93.0)	<0.001	185 (60.9)	165 (54.5)	0.111	24.20	6.70 (3.92-11.45)	<0.001
Diagnostic criteria for hypertension in adults	156 (51.5)	241 (79.8)	<0.001	186 (61.2)	195 (64.4)	0.419	25.10	3.33 (2.28-4.88)	<0.001
Whether high BP is related to smoking	229 (75.6)	239 (79.1)	0.330	223 (73.4)	228 (75.2)	0.594	1.70	1.08 (0.70-1.65)	0.734
Whether high BP is related to long-term drinking	232 (76.6)	241 (79.8)	0.374	239 (78.6)	242 (79.9)	0.704	1.90	1.10 (0.71-1.70)	0.677
Whether high BP is related to high salt intake	244 (80.9)	274 (90.7)	<0.001	244 (80.3)	241 (79.5)	0.824	10.60	2.46 (1.45-4.16)	<0.001
Whether high BP is related to overweight or obesity	223 (73.9)	226 (74.8)	0.780	206 (67.8)	214 (70.6)	0.445	-1.90	0.92 (0.59- 1.43)	0.698
Complications of hypertension									
*Coronary heart disease*	175 (57.8)	268 (88.7)	<0.001	194 (63.8)	181 (59.7)	0.301	35.00	7.28 (4.62-11.49)	<0.001
*Stroke*	230 (75.9)	160 (86.1)	0.001	262 (86.2)	258 (85.1)	0.728	11.30	2.17 (1.29-3.67)	0.004
*RDI of salt for adults*	194 (64.0)	286 (94.7)	<0.001	175 (57.6)	210 (69.3)	0.003	19.00	5.82 (3.36-10.07)	<0.001
**Attitude of hypertension prevention**									
Population at risk for hypertension should improve their lifestyle	202 (66.7)	251 (83.1)	<0.001	267 (87.8)	263 (86.8)	0.703	17.40	2.70 (1.67-4.37)	<0.001
Reducing salt intake can help prevent hypertension	252 (83.2)	257 (92.5)	0.516	245 (80.6)	244 (80.5)	0.984	9.40	1.16 (0.72-1.86)	0.543
Quitting smoking can help prevent hypertension	209 (69.0)	238 (78.8)	0.006	236 (77.6)	241 (79.5)	0.567	7.90	1.48 (0.97-2.25)	0.070
Reducing alcohol intake can help prevent hypertension	215 (71.0)	258 (85.4)	<0.001	246 (80.9)	255 (84.2)	0.294	11.10	1.93 (1.19-3.11)	0.008
Controlling body weight can help prevent hypertension	219 (72.3)	240 (79.5)	0.039	204 (67.1)	218 (71.9)	0.195	2.40	1.16 (0.72-1.85)	0.542
Population at risk for hypertension should monitor their BP	213 (70.3)	275 (91.1)	<0.001	256 (84.2)	254 (93.8)	0.898	11.20	4.56 (2.78-7.46)	<0.001
**Hypertension-related behaviours**									
Not smoking	267 (88.1)	276 (91.4)	0.185	234 (77.0)	232 (76.6)	0.906	3.7	2.50 (1.45-4.30)	0.001
Not drinking alcohol	262 (86.5)	262 (86.8)	0.918	235 (77.3)	229 (75.6)	0.617	2.0	2.02 (0.16-25.09)	0.586
Control of salt intake	262 (86.5)	263 (87.1)	0.823	224 (73.7)	223 (73.9)	0.946	0.4	1.05 (0.67-1.64)	0.826
Controlling body weight	153 (50.5)	212 (70.2)	<0.001	114 (37.5)	148 (48.8)	0.005	8.4	1.10 (0.69-1.76)	0.679
Frequent physical activity	167 (55.1)	193 (63.9)	0.028	100 (32.9)	107 (35.3)	0.530	6.4	1.51 (0.97-2.33)	0.066
Monitoring BP regularly	199 (65.4)	296 (98.0)	<0.001	148 (48.7)	141 (46.5)	0.596	34.8	29.61 (13.02-67.35)	<0.001

BP – blood pressure, RDI – recommended daily intake, OR – odds ratio, DID – difference-in-difference, CI – confidence interval

*P-values were calculated by χ^2^ tests.

†The models were adjusted for age, gender, educational level, and income.

The DID analyses indicated that the intervention measures were associated with a 24.2% increase in the knowledge awareness rates on “hypertension is a lifelong disease” (OR = 6.70, 95% CI = 3.92-11.45), a 25.1% increase in “the diagnostic criteria for hypertension” (OR = 3.33, 95% CI = 2.28-4.88), a 10.6% increase in “high BP is related to high salt intake” (OR = 2.46, 95% CI = 1.45-4.16), a 35.0% increase in “coronary heart disease is a complication of hypertension” (OR = 7.28, 95% CI = 4.62-11.49), an 11.3% increase in “stroke is a complication of hypertension” (OR = 2.17, 95% CI = 1.29-3.67), and a 19.0% increase in “RDI of salt for adults” (OR = 5.82, 95% CI = 3.36-10.07).

Regarding hypertension-related attitudes, there were significant differences in the rates of positive answers to all questions on attitudes before and after the intervention in the intervention group (all *P* < 0.05), except for “reducing salt intake can help prevent hypertension”. The DID results demonstrated that there were larger improvements in the increments of the belief formation rates in the intervention group than in the control group, regarding questions on “population at risk for hypertension should improve their lifestyle” (OR = 2.70, 95% CI = 1.67-4.37), “reducing alcohol intake can help prevent hypertension” (OR = 1.93, 95% CI = 1.19-3.11), and “population at risk for hypertension should monitor their BP” (OR = 4.56, 95% CI = 2.78-7.46).

Regarding the behaviour and lifestyle change, the proportions of those who were frequently physically active and monitoring BP regularly in the intervention group and the proportion of those controlling body weight in both groups were significantly improved at one-year follow-up. According to the DID analysis, the improvement of the proportion of those who were not smoking attributable to the intervention was 3.7% (OR = 2.50, 95% CI = 1.45-4.30) and 34.8% (OR = 29.61, 95% CI = 13.02-67.35) for those monitoring BP regularly.

## DISCUSSION

To our knowledge, this is the first community-based randomized controlled trial to assess the effects of the integrated programme in the management of the population at risk for hypertension in Eastern China. Our results showed that the community-based integrative programme could improve hypertension-related KAB and reduce the risk for hypertension in Eastern China in a one-year follow-up period.

Our findings indicate that the integrative programme could effectively reduce hypertension incidence in high-risk groups. A previous study conducted in Thailand has shown only marginally significant effects on delaying the onset of hypertension [15]. One major reason for our success was that the intervention package was a comprehensive and collaborative programme, led by trained GPs in conjunction with high-risk individuals. The intensive and strict follow-up by GPs and the regular self-monitoring by the participants were the key elements of the programme. Through scheduled follow-up visits, the GPs could interact with the high-risk populations, help them better understand health-related information [25], and persuade them to develop healthy lifestyles. By recording their physical activities, dietary patterns, smoking, and alcohol consumption once a month, participants could get a comprehensive understanding of their health status and behaviour patterns. Moreover, the intervention could strengthen their responsibility for their own health status, which would in turn help form healthy behaviours. When participants noticed that their BP fluctuated significantly, they were more likely to try to explore the reasons for the fluctuation according to their acquired knowledge and adjust their behaviours consciously [26]. Therefore, after a one-year intervention in our study, the individuals in the intervention group were more likely to improve their hypertension-related knowledge and adopt healthy behaviours than those in the control group, which all contributed to the reduction of their risks for hypertension [27]. Indeed, the hypertension incidence might also be affected by other factors such as the family history of hypertension, and lipid level, which we did not manage to measure and control in the analyses. However, given that this is a randomized trial, these factors were probably balanced out between the intervention and control groups. Therefore, the reduction of hypertension incidence should be mostly attributable to the integrative programme.

Our study indicated that the intervention helped improve the adoption of regular BP monitoring behaviour, comparable to a previous study’s findings [28]. After the intervention, the belief formation that “people at high risk of hypertension should monitor their BP” has improved significantly, while other behaviours were less formed, which could help the individuals take the initiative to monitor BP or do it more frequently. There may be several explanations for why measuring BP was more acceptable than the adoption of other behaviours. First, the notion that regular BP monitoring is one of the essential measures for preventing hypertension has been widely disseminated and accepted by the public for a long time [29]. Second, it takes minimum time and effort to monitor BP and has little impact on participants’ daily life. Most individuals at risk for hypertension were willing to prepare electronic sphygmomanometers at home to regularly record BP [30], which is easy to do, inexpensive, and accessible [31]. This easy-to-adopt behaviour should be recommended as one of the priorities during the implementation of the intervention, boosting the participants' confidence in the behaviour intervention, leading to improvements. However, it is unclear whether the participants would keep the habit of monitoring BP or stop doing it in a short period. Therefore, it would be important to establish a community-based health promotion environment for retaining the frequency of BP monitoring, involving family members to remind and supervise them to measure BP regularly.

We also observed the discrepancies of KAB on the same topic. The awareness rate of “whether high BP is related to high salt intake” and “RDI of salt for adults” increased significantly in the intervention group, but there was no significant improvement in the behaviour formation of controlling salt intake. The belief that “reducing alcohol intake can help prevent hypertension” improved significantly, but the behaviour of alcohol intake did not decrease. Such discrepancies are likely due to the fact that the pathways between knowledge and behaviour change can be influenced by many other factors [32], such as difficulty in changing habits or lack of environmental support. It has been recognized that it is usually more acceptable to develop new behaviours than to change or give up on existing ones, which is a big challenge to the improvement of healthy behaviour. For instance, it would take time and effort to shift from salty food consumption to a light diet due to personal preferences, other family members’ influence, social norms, and culture [33]. Therefore, more emphasis and effort should be placed on changing unhealthy behaviour, possibly through environmental support, community engagement, and personal skill enhancement. Participants who have formed healthy beliefs could be guided into the process of “try – start – persist” of developing healthy behavioural habits. For example, to help the population at risk for hypertension engage in physical exercises, sessions about how to exercise appropriately should be widely promoted, and exercising groups should be encouraged and organized [34] so that individuals can help each other and gradually develop habits of regular physical activities. Moreover, family members and GPs should guide, motivate, and support high-risk groups by appreciating and encouraging them when they progress in modifying their behaviours [31].

This study has several limitations. First, due to the outbreak of COVID-19 pandemic during the intervention implementation, some intervention measures (eg, offline health education lectures) could not be conducted effectively, which may have affected the programme’s net effects. Second, health behaviours were mainly self-reported without using scales, which may have led to some reporting bias. Third, a double-blind procedure was not applicable to our study, because the researchers needed to communicate with the GPs about the intervention protocol and process. The data collection and data analysis were all objectively implemented by the GPs and the researchers so as to get reliable results. Fourth, we used a cluster randomized sampling to select the participants with balanced demographic characteristics between groups, while the prevalence of hypertension-related (healthy) behaviours at baseline substantially differed between groups. Therefore, we adjusted for baseline KAB when analysing the effect on the incidence of hypertension and used the DID analyses to assess the intervention’s net effects on KAB. Finally, the study only included 6 neighbourhood blocks as clusters and only assessed short-term effects (12 months post-intervention), meaning some of the intervention effects may not be revealed. A longer-term assessment (18 or 24 months) and a trial with a larger number of clusters are needed to further examine the effectiveness of the integrative programme.

## CONCLUSIONS

We found that the community-based integrative program could reduce the risk of hypertension and improve hypertension-related KAB in the communities of Eastern China. The intervention could encourage the development of new healthy behaviours, but had little effect on the modification of unhealthy ones. Future intervention strategies should be targeted to change unhealthy lifestyles with more incentive measures. The community-based management of the population at risk should be scaled up to reach all communities throughout the country, which would reduce the individual and societal burden of hypertension and cardiovascular disease in China.
